# Supplementary material for: IL11 Stimulates IL33 Expression and Proinflammatory Fibroblast Activation across Tissues
Source: Int J Mol Sci. 2022 Aug 10;23(16):8900. doi: 10.3390/ijms23168900 (PMC9408968; doi:10.3390/ijms23168900)
Supplement: Supplementary file 1 [file ijms-23-08900-s001.zip › ijms-1832112-supplementary.pdf]

Supplementary Materials

Supplementary Table S1. Differentially expressed genes in human kidney fibroblasts (HKF) stimulated with IL11 for 1, 6, and 24 h as compared to unstimulated HKF. Data was sorted by log2 (fold change) and adjusted p-values. Genes with base mean values of 0 were excluded.

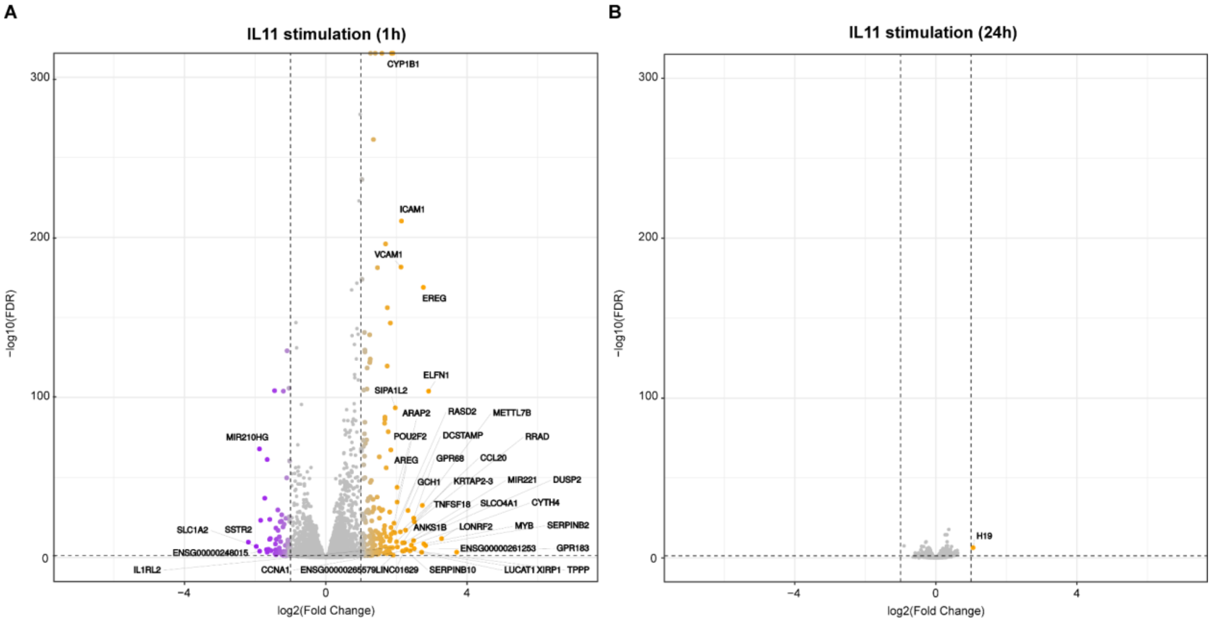

Figure S1. RNA sequencing of IL11-stimulated human kidney fibroblasts. Volcano plots displaying fold change (FC) of significantly differentially expressed genes between baseline and IL11 (10 ng/ml)-stimulated HKFs at 1h (**A**) and 24h (**B**) time points. Dashed lines are drawn to define the log2 FC value of 1 (vertical) and -log10 FDR of 0.05 (horizontal). Upregulated, downregulated, and non-differentially expressed genes are labeled in orange, purple, and gray, respectively.

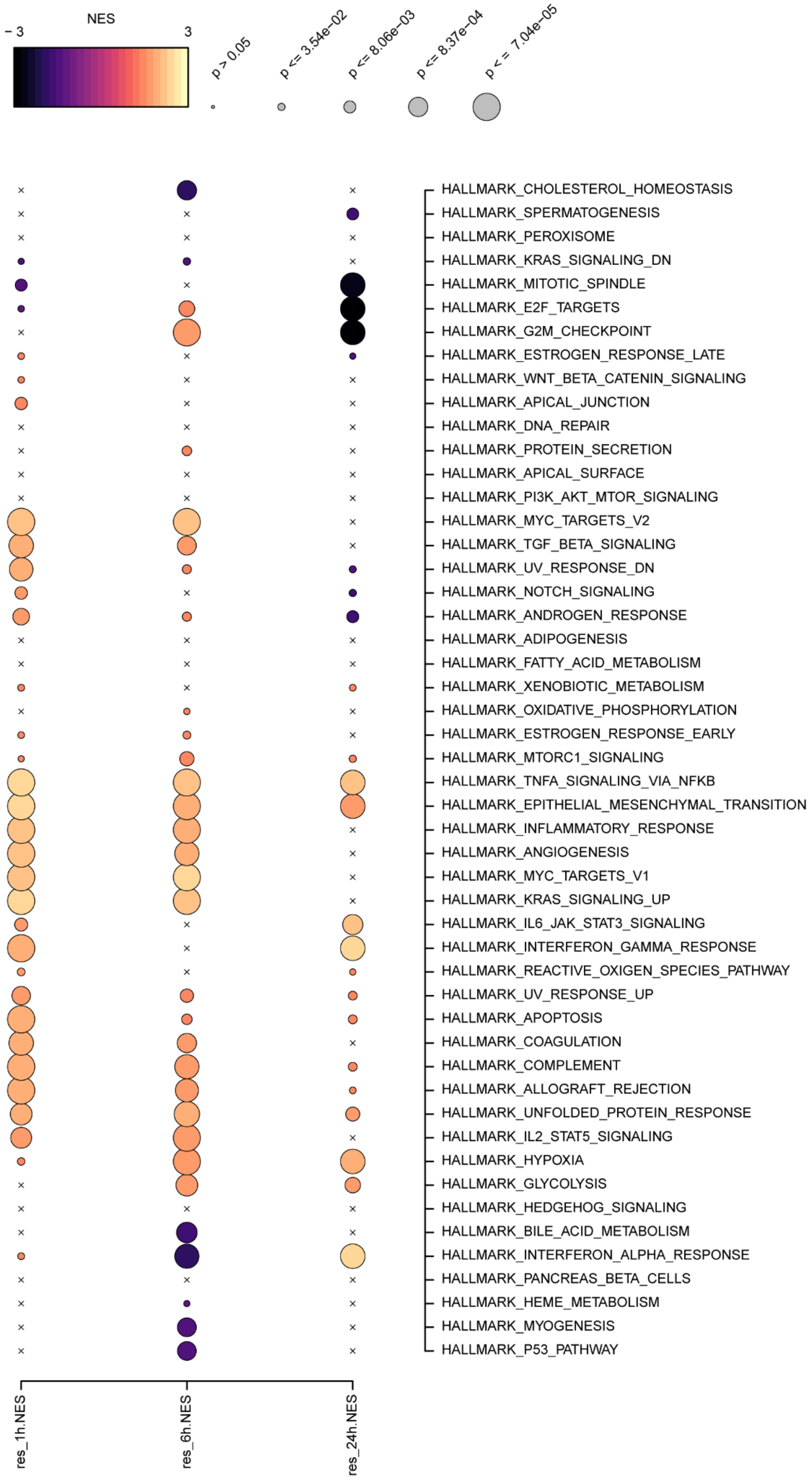
NES
− 3
3
p > 0.05
p <= 3.54e−02
p <= 8.06e−03
p <= 8.37e−04
p < = 7.04e−05
HALLMARK_CHOLESTEROL_HOMEOSTASIS
HALLMARK_SPERMATOGENESIS
HALLMARK_PEROXISOME
HALLMARK_KRAS_SIGNALING_DN
HALLMARK_MITOTIC_SPINDLE
HALLMARK_E2F_TARGETS
HALLMARK_G2M_CHECKPOINT
HALLMARK_ESTROGEN_RESPONSE_LATE
HALLMARK_WNT_BETA_CATENIN_SIGNALING
HALLMARK_APICAL_JUNCTION
HALLMARK_DNA_REPAIR
HALLMARK_PROTEIN_SECRETION
HALLMARK_APICAL_SURFACE
HALLMARK_PI3K_AKT_MTOR_SIGNALING
HALLMARK_MYC_TARGETS_V2
HALLMARK_TGF_BETA_SIGNALING
HALLMARK_UV_RESPONSE_DN
HALLMARK_NOTCH_SIGNALING
HALLMARK_ANDROGEN_RESPONSE
HALLMARK_ADIPOGENESIS
HALLMARK_FATTY_ACID_METABOLISM
HALLMARK_XENOBIOTIC_METABOLISM
HALLMARK_OXIDATIVE_PHOSPHORYLATION
HALLMARK_ESTROGEN_RESPONSE_EARLY
HALLMARK_MTORC1_SIGNALING
HALLMARK_TNFA_SIGNALING_VIA_NFKB
HALLMARK_EPITHELIAL_MESENCHYMAL_TRANSITION
HALLMARK_INFLAMMATORY_RESPONSE
HALLMARK_ANGIOGENESIS
HALLMARK_MYC_TARGETS_V1
HALLMARK_KRAS_SIGNALING_UP
HALLMARK_IL6_JAK_STAT3_SIGNALING
HALLMARK_INTERFERON_GAMMA_RESPONSE
HALLMARK_REACTIVE_OXIGEN_SPECIES_PATHWAY
HALLMARK_UV_RESPONSE_UP
HALLMARK_APOPTOSIS
HALLMARK_COAGULATION
HALLMARK_COMPLEMENT
HALLMARK_ALLOGRAFT_REJECTION
HALLMARK_UNFOLDED_PROTEIN_RESPONSE
HALLMARK_IL2_STAT5_SIGNALING
HALLMARK_HYPOXIA
HALLMARK_GLYCOLYSIS
HALLMARK_HEDGEHOG_SIGNALING
HALLMARK_BILE_ACID_METABOLISM
HALLMARK_INTERFERON_ALPHA_RESPONSE
HALLMARK_PANCREAS_BETA_CELLS
HALLMARK_HEME_METABOLISM
HALLMARK_MYOGENESIS
HALLMARK_P53_PATHWAY
res_1h.NES
res_6h.NES
res_24h.NES

Figure S2. IL11 induces a pro-inflammatory transcriptional response in human kidney fibroblasts. Bubblemap showing results of hallmark gene set enrichment analysis (GSEA) for differentially expressed genes following IL11 stimulation over a time course (0, 1, 6, and 24 h). Each dot represents the normalized enrichment score (NES) for the gene set and its FDR-corrected significance level, summarized by colour and size respectively. Gene sets for the enrichment test were selected from the "H - Hallmark" collection in MSigDB.
