# Supplementary figures and images for: IL11 Stimulates IL33 Expression and Proinflammatory Fibroblast Activation across Tissues
Source: Int J Mol Sci. 2022 Aug 10;23(16):8900. doi: 10.3390/ijms23168900 (PMC9408968; doi:10.3390/ijms23168900)

Figure 1A

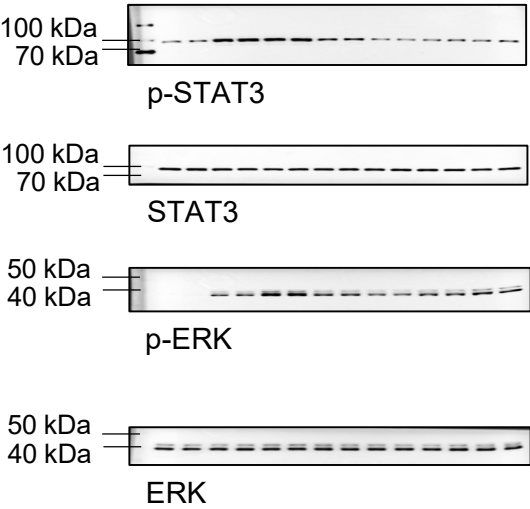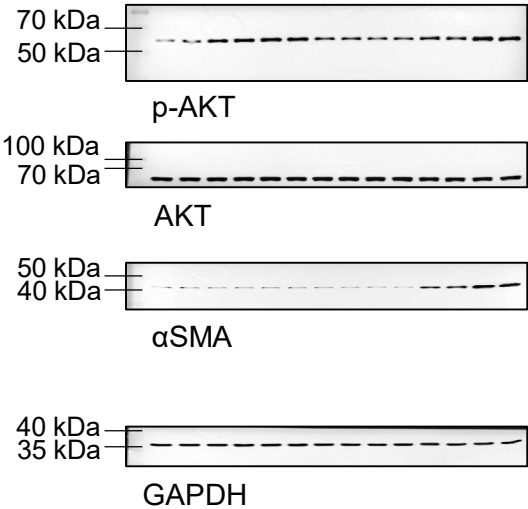

Figure 1B

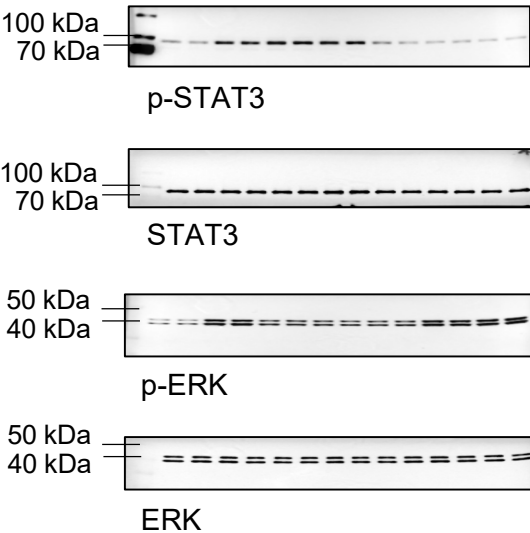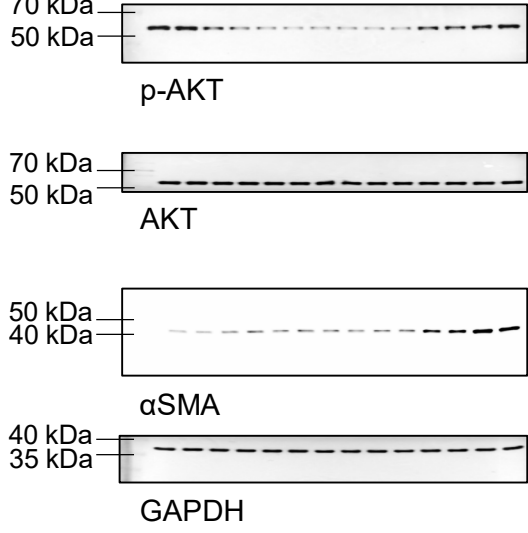

Figure 1C

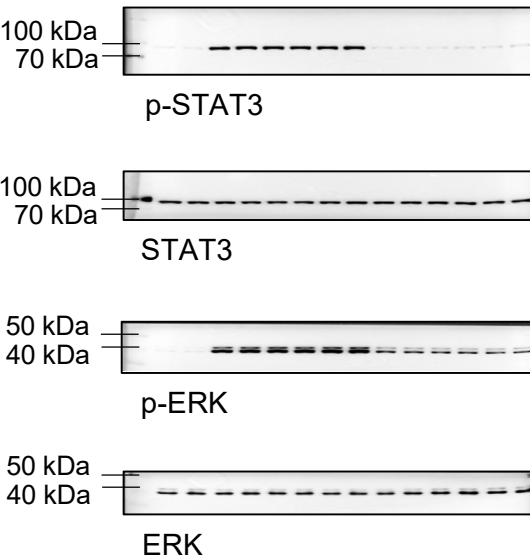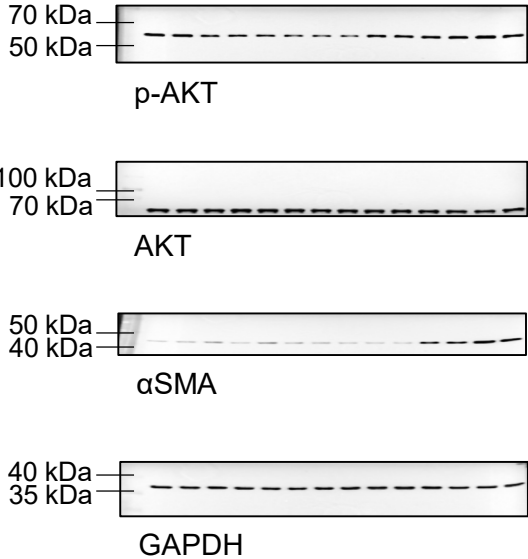

Figure 4A

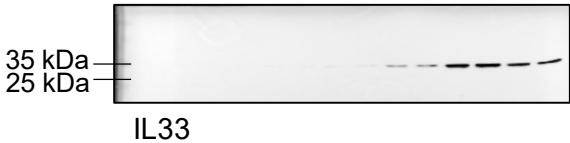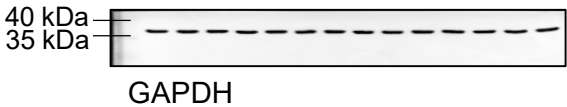

Figure 4B

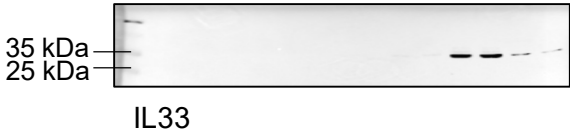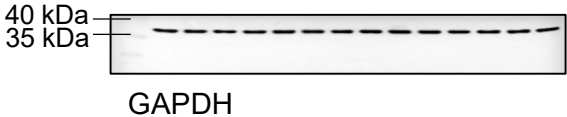

Figure 4C

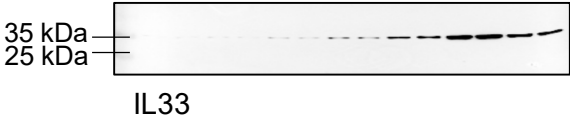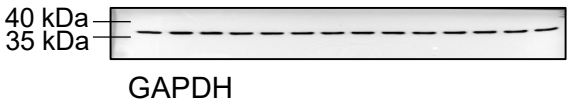

Figure 4D

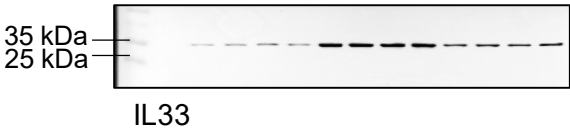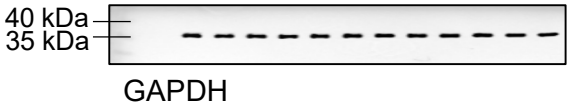

Supplement: Supplementary file 1 [file ijms-23-08900-s001.zip › Datafile S1.pdf]
